# Understanding dual process cognition via the minimum description length principle

**DOI:** 10.1371/journal.pcbi.1012383

**Published:** 2024-10-18

**Authors:** Ted Moskovitz, Kevin J. Miller, Maneesh Sahani, Matthew M. Botvinick

**Affiliations:** 1 Gatsby Computational Neuroscience Unit, University College London, London, United Kingdom; 2 Google DeepMind, London, United Kingdom; 3 Department of Ophthalmology, University College London, London, United Kingdom; Scuola Internazionale Superiore di Studi Avanzati, ITALY

## Abstract

Dual-process theories play a central role in both psychology and neuroscience, figuring prominently in domains ranging from executive control to reward-based learning to judgment and decision making. In each of these domains, two mechanisms appear to operate concurrently, one relatively high in computational complexity, the other relatively simple. Why is neural information processing organized in this way? We propose an answer to this question based on the notion of compression. The key insight is that dual-process structure can enhance adaptive behavior by allowing an agent to minimize the description length of its own behavior. We apply a single model based on this observation to findings from research on executive control, reward-based learning, and judgment and decision making, showing that seemingly diverse dual-process phenomena can be understood as domain-specific consequences of a single underlying set of computational principles.

## Author summary

Since the founding of psychology as a scientific discipline, scientists have recognized that much of human and animal behavior falls into two separate categories: slow, effortful deliberation (e.g., solving math problems) and fast, automatic habitual action selection (e.g., signing your name). Such “dual process” theories of cognition have since been developed to explain a number of phenomena from across psychology and neuroscience. Despite all of these experimental results, understanding *why* this pattern in behavior exists has proven elusive. In this work, we develop a model of dual process cognition which can be derived independently from experimental observations about the brain. Our theory proposes that the division between effortful and automatic behavior emerges as a natural result of the need to balance mastery of the tasks we have to complete every day and flexibility to adapt to new circumstances. In a large number of computer simulations, our model captures many behavioral phenomena associated with “dual process” cognition across multiple subfields of cognitive science.

## Introduction

William James famously distinguished between two modes of action selection, one based on habit and the other involving effortful deliberation [1]. This idea has since ramified into a variety of “dual-process” theories in at least three distinct domains of psychology and neuroscience. One of these domains concerns executive control, and distinguishes action selection that is “automatic”, reflecting robust stimulus-response associations, from that which is “controlled”, overriding automatic actions when necessary [2, 3]. A second focuses on reward-based learning, distinguishing behavior that is sensitive to current goals (“goal-directed” or “model-based”) from that which is habitual [4, 5]. The third addresses judgment and decision making (JDM), where canonical theories distinguish between two cognitive systems: a “System 1”, which employs fast and frugal heuristic decision strategies, and a “System 2”, which supports more comprehensive reasoning [6, 7].

While the reduction of action selection to dual processes is undoubtedly a simplification, across these three domains, dual-process models have accumulated considerable empirical support, and each domain has developed explicit computational models of how dual processes might operate and interact [3, 5, 8–14]. These computational models, however, are typically domain-specific, reproducing behavioral phenomena that are within the scope of their domain. It remains unknown whether dual-process phenomena in different domains result from different sets of computational mechanisms, or whether they can be understood as different manifestations of a single, shared set. That common mechanisms might be at play is suggested by a wealth of neuroscientific data. Specifically, studies have linked controlled behavior, model-based action selection, and System-2 decision making with common circuits centering on the prefrontal cortex [2, 4, 15–18], while automatic behavior, habitual action selection, and heuristic decision making appear to engage shared circuits lying more posterior and running through the dorsolateral striatum [18–21]. While further study into these neuroanatomical relationships is required, these results do beg the question of whether a single computational model could account for these patterns of decision-making.

In this work, we add to the growing body of literature which seeks a *normative explanation* for these phenomena [22–26]. That is, we seek a theory that can reproduce behavioral findings associated with dual process cognition, but which is derived instead from an optimization principle, allowing dual process cognition to be understood as the solution to a fundamental behavioral or computational problem. To identify such a principle, we begin by considering a fundamental problem confronting both biological and machine intelligence: generalization. We discuss a fundamental computational theory of generalization, link it to behavior, and demonstrate that a recently-proposed behavioral model from machine learning based on this principle can successfully reproduce canonical dual-process phenomena from executive control, reward-based learning, and JDM.

### Computational principle: Generalization via compression

A fundamental demand of intelligent behavior is to capitalize on past learning in order to respond adaptively to new situations, that is, to generalize. Humans in particular show a remarkable capacity for behavioral generalization, to such a degree that this has been regarded as one of the hallmarks of human intelligence [27]. From a modeling perspective, one way to generalize is to capture shared structure underlying the tasks with which a decision-maker is faced. However, if a model has too many degrees of freedom, it can *overfit* to noise in the observed data which may not reflect the true distribution. In approaching this problem, the machine learning literature points consistently to the importance of *compression*: In order to build a system that effectively predicts the future, the best approach is to ensure that that system accounts for past observations in the most compact or economical way possible [28–31]. This Occam’s Razor-like philosophy is formalized by the *minimum description length* (MDL) principle, which prescribes finding the shortest solution written in a general-purpose programming language which accurately reproduces the data, an idea rooted in *Kolmogorov complexity* [32]. Given that actually computing Kolmogorov complexity is impossible in general, MDL theory advocates for a more practical approach, proposing that the best representation or model *M* for a body of data *D* is the one that minimizes the expression

$$\begin{array}{c} L(M)+L(D|M). \end{array}$$ (1)

*L*(*M*) here is the description length of the model, that is, the number of bits it would require to encode that model, a measure of its complexity [30]. *L*(*D*|*M*), meanwhile, is the description length of the data given the model, that is, an information measure indicating how much the data deviates from what is predicted by the model. In short, MDL favors the model that best balances between deviation and complexity, encoding as much of the data as it can while also remaining as simple as possible.

In order to practically model these codes, one can choose from a number of *universal coding* schemes, so-called because they are guaranteed to behave monotonically with respect to the true underlying code lengths. One such encoding scheme is the *variational code* [33–35], which implements the deviation cost via the negative log-likelihood of the data under the model and the complexity cost as the Kullback-Leibler (KL) divergence between the model and a sparse base distribution. Minimizing this objective is equivalent to performing variational inference with a particular choice of simplicity-inducing prior. While there are many such universal coding schemes [35, 36], we focus on the variational code in this work due to its compatibility with neural network implementation.

### Minimum description length control (MDL-C)

Given a normative principle for generalization, the next step in developing our model is to apply the MDL principle in the context of decision-making. This means defining an ‘agent’ that receives observations of the environment and emits actions based on an adjustable ‘policy,’ a mapping from situations to actions. A ‘task’ is defined as a combination of an environment and some objective that the agent’s policy is optimized to accomplish within that environment. The MDL principle holds that *learning* is the process of discovering regularity in data, and that any regularity in the data can be used to *compress* it [37]. In order to apply MDL theory to an agent, then, we must define what exactly the “data” that we want to compress. [38] propose that agents faced with a multitude of tasks should aim to identify common behavioral patterns that arise in the solutions to these tasks. In other words, the data that the agent should seek to compress are useful patterns of interaction with the world—optimal policies—for solving the problems it most frequently faces. To align with MDL theory, a behavioral system for generalization needs to accomplish two objectives. First, it must generate data by solving tasks, and second, it must identify useful structure in these data through compression. These objectives are assigned to two processes: a behavioral, or “control” policy *π*, which aims to find solutions to new tasks, and an auxiliary, or “default” policy *π*_0_, which attempts to compress these solutions.

This principle is applicable to any behaviorally-defined objective function (e.g. imitation learning [39]). In our simulations, we consider objective functions defined via the reinforcement learning framework (RL; [40]), in which the environment delivers quantitative ‘rewards’ in way that depends on its state and on the agent’s actions, and the agent attempts to maximize these rewards. This framework is appealing for modeling behavior in tasks from multiple disciplines, as it assumes no *a priori* access to a model of the world, generalizes a number of other learning paradigms (e.g., any supervised learning problem can be cast as an RL task), and can be adapted to both simple and complex observation types via function approximation. These objectives can be combined in the following expression:

$$\begin{array}{c} E_{\pi }\left[R\right]-\lambda \left[L\left(\pi_{0}\right)+L\left(\pi |\pi_{0}\right)\right], \end{array}$$ (2)

where *R* denotes cumulative reward and λ as a weighting parameter. Maximizing this objective yields a form of regularized policy optimization which [38] call *minimum description length control* (MDL-C). At an intuitive level, MDL-C trains the learning agent to formulate a policy that maximizes reward while also staying close to a simpler or more compressed reference policy. By compressing useful behavioral patterns from past experience, this default policy can guide the control policy to more quickly find solution to new tasks [38]. This division of the agent into two modules, one of which is incentivized to solve new tasks and the other to compress those solutions, is reminiscent of the many dual-process theories in psychology and neuroscience. Crucially, this organization is here derived from first-principles reasoning about the requirements of combining the MDL principle with adaptive behavior, rather than neuroscientific or psychological data.

**Fig 1:**
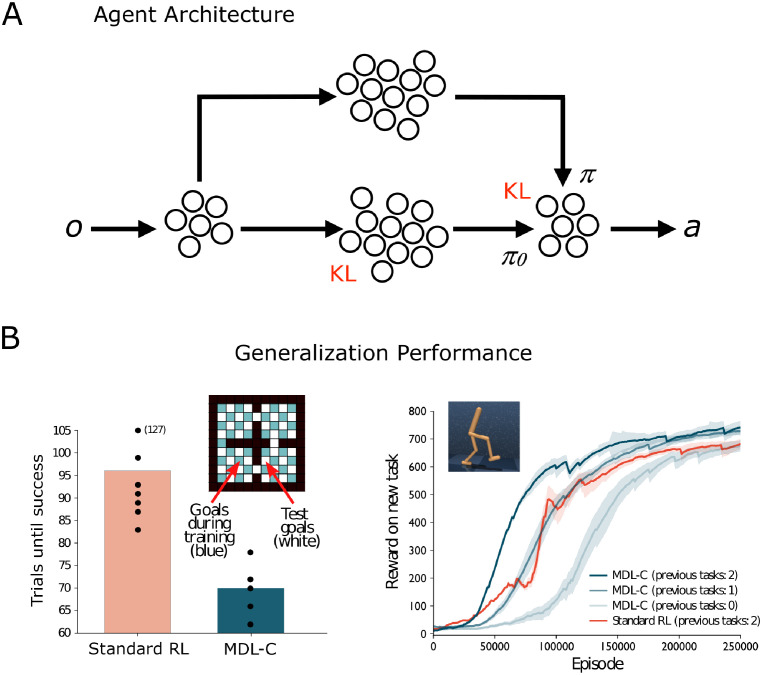
Schematic of MDL-C implementation and generalization results. A: Neural network implementation of MDL-C. Perceptual observations (input *o*) feed into two recurrent networks. The lower pathway ($RNN_{\pi_{0}}$) has noisy connections with VDO regularization, outputting action distribution *π*_0_. The upper pathway (*RNN*_*π*_) outputs distribution *π*, which overwrites *π*_0_. KL divergence between policies is computed, and action *a* is selected from *π*. B: MDL regularization enhances generalization. Left: MDL-C agent vs unregularized baseline (Standard RL) in grid navigation task. Barplot shows average trials to find shortest path to new goals. Right: Average reward in continuous control task. MDL-C learns faster with related task experience and outperforms Standard RL.

Recent advances in artificial intelligence (AI) allow us to implement MDL-C in the form of a runnable simulation model, as diagrammed in Fig 1 (see S1 Methods). Here, both policy *π* and policy *π*_0_ are parameterized as identical recurrent neural networks, both receiving the same perceptual inputs. On every time-step, the network implementing the reference policy *π*_0_—henceforth $RNN_{\pi_{0}}$—outputs a probability distribution over actions. That distribution is then updated by the network implementing policy *π* (*RNN*_*π*_), and the agent’s overt action is selected (see S1 Methods). To implement MDL regularization using a variational code, the deviation term *L*(*π*|*π*_0_) is quantified as the KL divergence between the two policies *π* and *π*_0_, consistent with the fact that the KL divergence represents the amount of information required to encode samples from one probability distribution (here *π*) given a second reference distribution (*π*_0_). In order to implement the complexity cost *L*(*π*_0_), we apply a technique known as variational dropout [41]. VDO applies a form of multiplicative Gaussian noise to the network activations which is equivalent to applying a KL divergence penalty between the distribution over model weights and a sparse prior. There are multiple possible choices for such a prior, but we apply the *Jeffreys prior* [42], which, in conjunction with a policy distribution in the exponential family, is asymptotically equivalent to the *normalized maximum likelihood estimator*, perhaps the most fundamental MDL estimator [43]. For more details, see the S1 Methods section and [38].

Equipped with this runnable implementation, we can return to the problem of generalization, and ask whether MDL-C in fact enhances generalization performance. In other words, we’d like to verify that this regularization enables the agent to adapt more quickly than it would otherwise to new goals. Fig 1B and 1C presents relevant simulation results (see also S1 Methods, and [38] for related theoretical analysis and further empirical evaluation). When our MDL-C agent is trained on a set of tasks from a coherent domain (e.g., navigation or gait control) and then challenged with a new task from this same domain, it learns faster than an agent with the same architecture but lacking MDL regularization. In short, policy compression, following the logic of MDL, enhances generalization. For further examples, see [38].

Having established these points, we are now in position to advance the central thesis of the present work: We propose that MDL-C may offer a useful normative model for dual-process behavioral phenomena. As in dual-process theory, MDL-C contains two distinct decision-making mechanisms. One of these (corresponding to $RNN_{\pi_{0}}$ in Fig 1A) distills as much target behavior as possible in an algorithmically simple form, reminiscent of the habit system or System 1 in dual-process theory. Meanwhile, the other (*RNN*_*π*_) enjoys greater computational capacity and intervenes when the simpler mechanism fails to select the correct action, reminiscent of executive control or System 2 in dual-process theory. MDL-C furnishes a normative explanation for this bipartite organization by establishing a connection with the problem of behavioral generalization. To test whether MDL-C can serve as such a model, we conducted a series of simulation studies spanning the three behavioral domains where dual-process theory has been principally applied: executive control in Simulation 1, reward-based decision making in Simulation 2, and JDM in Simulation 3.

#### General methods: Selection of target phenomena and approach to modeling

A detailed description of simulation methods, sufficient to fully replicate our work, is presented in S1 Methods. Briefly, for each target dual-process domain, we focused on a set of empirical phenomena that the relevant specialty literature treats as fundamental or canonical. We do not, of course, address all behavioral and neural phenomena that might be considered relevant to constrain theory in each domain, and we dedicate a later section to the question of whether any empirical findings that we do not directly model might present challenges for our theory. Nevertheless, the core phenomena in each field are fairly well recognized, and we expect our selections will be uncontroversial. Indeed, each target phenomenon has been the focus of previous computational work, and we dedicate a later section to comparisons between our modeling approach and previous proposals. While such comparisons are of course important, one point that we continue to stress throughout is that no previous model has addressed the entire set of target phenomena, bridging between the three domains we address.

For each target phenomenon, we pursue the same approach to simulation: We begin with a generic MDL-C agent model, configured and initialized in the same way across simulations (with the exception of input and output unit labels tailored to the task context). The model is then trained on an appropriate target task and its behavior or internal computations queried for comparison with target phenomena. Importantly, the model is in no case directly optimized to capture target phenomena, only to solve the task at hand. In the rare case where target effects depend sensitively on experimenter-chosen hyperparameters of MDL-C, this dependency is described alongside other results.

While our simulations focus on target phenomena that have been documented across many experimental studies, in presenting each simulation we focus on observations from one specific (though representative) empirical study, to provide a concrete point of reference. It should be noted that the target phenomena we address, in almost all cases, take the form of qualitative rather than quantitative patterns. Our statistical tests, described in S1 Methods, thus take the form of qualitative hypothesis tests rather than quantitative fits to data, paralleling the reference experimental research.

### Results

#### Simulation 1: Executive control

As introduced above, longstanding theories of executive function center on a contrast between two kinds of action. Habitual or automatic responses are default, reactive actions, shaped by frequency or practice. Controlled responses, in contrast, take fuller account of the task context, overriding automatic responses when they are inappropriate [2, 3, 17]. Some of the strongest support for this distinction comes from studies of prefrontal cortex. Prefrontal neural activity has been shown to play a special role in encoding goals, task instructions, and other aspects of task context [2, 17]. The importance of these representations for context-appropriate behavior is evident in the effects of prefrontal damage, where behavior tends to default to frequently performed actions, neglecting verbal instructions or context-appropriate goals.

**Fig 2:**
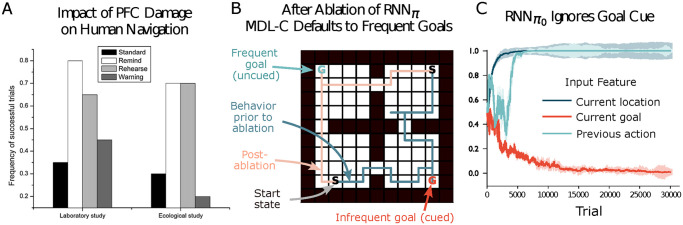
**A**. [44] reported that damage to another (orbitofrontal) region of PFC impaired navigation to novel goals, both in the laboratory and an ecological study. In unsuccessful trials patients frequently navigated to familiar goal locations. Performance improved when patients were given frequent reminders of the goal or were asked to verbally rehearse the goal, but not when the goal reminder was replaced by an uninformative stimulus (*Warning*). **B**. In a modified navigation task only two goals were cued, one (blue *G*) occurring more frequently during training than the other (red *G*). When the infrequent goal is cued at test, the intact MDL-C agent navigates successfully to it from any start state (see blue example trajectories). When *RNN*_*π*_ is ablated, the agent ignores the instruction cue and navigates to the more frequent goal (pink trajectories). See S1 Methods for simulation details. **C**. By inserting a gating layer over input features within $RNN_{\pi_{0}}$ (see S1 Methods), we can directly read out which information is processed by that pathway. The plot shows attention weights for the three input features in the navigation task referenced in Fig 1. Over the course of the initial training block, $RNN_{\pi_{0}}$ learns to ignore the current goal cue.

One domain in which these effects can be observed in a particularly straightforward form is spatial navigation. Prefrontal damage impairs the ability to navigate to instructed goal locations, with behaviour defaulting to more familiar paths and destinations [44] (Fig 2A).

Strikingly similar effects arise when MDL-C is applied to spatial navigation. In our first simulation, the MDL-C agent from Fig 1 was trained on a navigation task involving two cued goal locations, with one goal presented more frequently than the other (see S1 Methods). After training, *RNN*_*π*_ was able to successfully navigate to either goal when cued. However, when *RNN*_*π*_ was removed from the agent and it was forced to act using $RNN_{\pi_{0}}$—in a rough approximation of the PFC damage suffered by the patients studied by [44]—agents only ever navigated to the goal location that had been more frequently cued during training (Fig 2B). To gain a mechanistic understanding of why this occurs, we inserted a gating layer over inputs in $RNN_{\pi_{0}}$ to monitor which information is transmitted to the policy. We found that, despite the fact that both *RNN*_*π*_ and $RNN_{\pi_{0}}$ receive the same inputs, VDO induced $RNN_{\pi_{0}}$ to ignore the goal cue during training, as due to the difference in goal presentation frequencies, it was less predictive of *RNN*_*π*_’s behavior than other features.

**Fig 3:**
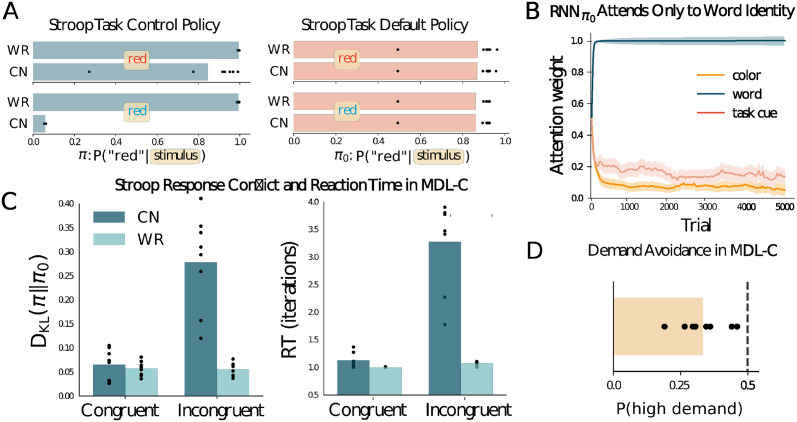
**A**. Policies for *RNN*_*π*_ (top) and $RNN_{\pi_{0}}$ (bottom) for the stimuli shown, in word-reading (*WR*) and color-naming (*CN*) contexts. Response probabilities are shown for the response *red*, complementary to (unshown) probabilities for the alternative *blue* response. **B**. When the MDL-C agent is trained on the Stroop task (see S1 Methods), $RNN_{\pi_{0}}$ learns to ignore both the task cue and the stimulus color, attending only to word identity. **C**. Left: KL divergence between *π* and *π*_0_ for the four trial types shown in panel A. Right: Corresponding reaction times (see S1 Methods). **D**. When trained on the Stroop task and then given a choice between blocks of color-naming trials that involve either high or low proportions of incongruent stimuli (see S1 Methods), the MDL-C agent displays a preference for less frequent incongruence, paralleling the demand-avoidance effect seen in human decision making.

To evaluate the generality of these effects, we applied MDL-C to another classic executive control problem, the Stroop task [45] (see S1 Methods and Fig 3). Here, words that name colors are presented in hues that are either incongruent (e.g., *RED* presented in blue) or congruent (*RED* in red). An instruction cue indicates whether the current task is to read the word, the highly practiced, automatic response, or to name the color, requiring cognitive control.

Consistent with the navigation results, while the control policy correctly learned to respond to both word-reading and color-naming trials (the former being presented more frequently in training), the default policy learned a simpler stimulus-response mapping based only on the written word (Fig 3A). These habit-like responses are overridden (by policy *π*) only when the task context requires it. When examining feature sensitivity, $RNN_{\pi_{0}}$, as in navigation, ignores the task context and is biased toward the behaviors executed most frequently during learning, consistent with the classical definition of automatic processing (Fig 3B).

Perhaps the defining behavioral phenomenon associated with the Stroop task is delayed reaction times on incongruent color-naming trials (as people are more used to reading words than naming colors) [3, 46], another finding replicated by MDL-C. MDL-C provides a simple way to reason about this pattern: because the control policy is regularized towards the default policy—which disagrees with the control policy on these inputs—its output distribution is less concentrated over the correct output, requiring more recurrent cycles to reach the response threshold. The KL divergence between the control and default policies was therefore highest for color-naming conflict trials, as it was in these trials alone for which simply matching the written word resulted in the incorrect response (Fig 3C). In this way, MDL-C provides a direct relationship between reaction time and the cost of control.

Another core phenomenon in the cognitive control literature is *demand avoidance*, the tendency for decision makers to avoid tasks that require intensive cognitive control [47]. For example, when human participants are asked to select between two versions of the Stroop task, one involving more frequent incongruent trials than the other, they show a clear tendency to avoid the former task and the demands on cognitive control it involves [48]. When MDL-C is trained in the same task context (see S1 Methods), the same choice bias arises (Fig 3D). The explanation for this result is tied to the final term in the MDL-C objective function (see Eq 2), which penalizes conflict between policies *π* and *π*_0_ (compare [25, 49]). By avoiding control-demanding tasks, the agent can minimize this term, helping it to minimize the description length of its overall behavioral policy.

The relation of the above simulation results to those from previous models, and a consideration of a wider range of empirical phenomena in the domain of executive control, are discussed below under *Comparison with previous models*.

#### Simulation 2: Reward-based learning

**Fig 4:**
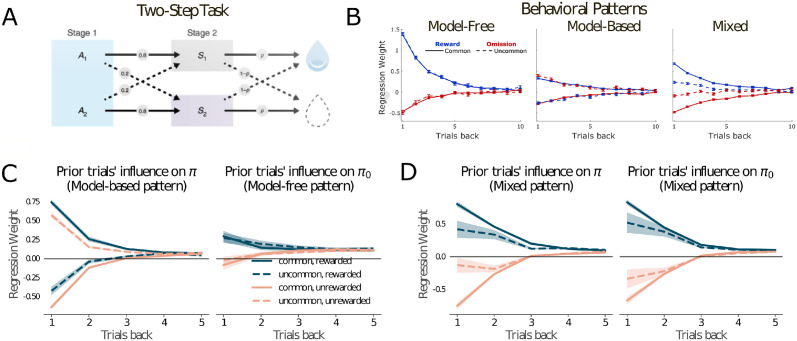
**A**. Structure of the two-step task as introduced by [50]. Choice occurs at Stage 1. The value of *p* varies over time, and so must be inferred by the participant. Following subsequent research, the version employed in our experiments additionally included explicitly cued reversals in the structure of transitions from Stage 1 to Stage 2. See S1 Methods for full details. **B**. Classical behavioral signatures of model-free (left) and model-based (center) performance in the two-step task. Adapted from [51], the plots show logistic regression weights quantifying the influence of two factors on the probability of repeating on the index trial the same first-stage action selected on the previous trial: (1) whether reward was received or omitted on the previous trial, and (2) whether the previous trial featured a transition from stage 1 to 2 that was high-probability (*common*) or low (*uncommon*). The right panel shows a hybrid pattern, similar to that reported in the classic study by [50]. **C**. Left: Two-step behavior of MDL-C, reflecting policy *π*. Right: Influence of the past on policy *π*_0_. **D**. Same as Panel D but with different weighting of terms in the MDL-C objective (see S1 Methods and compare panel C, right).

According to prevailing theories, reward-based learning centers on two distinct neural systems. One, operating within parts of prefrontal cortex and associated basal ganglia circuits, implements a “goal-directed” or “model-based” algorithm, which takes task structure into account. The other system, more posterior or lateral, operates in a “habitual” manner, based on simpler stimulus-response associations [4, 9, 14, 52–56]. Although the anatomical substrates proposed for these systems can resemble those associated with controlled and automatic processing, different behaviors have been used to study them. In research with humans, the most prominent of these is the so-called “two-step task” [50], illustrated in Fig 4A.

The two-step task was designed to probe the operation of model-based and habitual systems, under the hypothesis that these operate in parallel and that the habitual system implements model-free reinforcement learning [54, 55] (see *Comparison with previous models* and S1 Methods). In this task, subjects must choose between two options that will probabilistically transition them to one of two second stage states which themselves stochastically either produce reward or nothing (Fig 4A). According to the logic of the task, if the agent is able to learn a model of this transition structure, its policy update on the first step will be sensitive both to second-step reward as well as to whether the second-step state was the “common” or “uncommon” one given first-step action. This ability is reflected in behavioral patterns classically thought-of as diagnostic for model-based and model-free behavior on this task (Fig 4B), which shows the results of logistic regression from previous trial results to predict whether subjects repeated their most recent stage 1 choice. Synthetic behavioral data from a model-free (TD(1)) agent is associated with positive regression weights for trials which resulted in reward after both common and uncommon transitions, indicating a lack of understanding of the task structure. In contrast, synthetic behavioral data from a model-based agent is associated with positive regression weights for common, rewarded trials and uncommon, unrewarded trials. We trained MDL-C on a modified version of the task, in which the first stage transition probabilities also occasionally switch [58] (see S1 Methods for details), which increases the difference in computational complexity needed to exhibit the canonical model-based vs model-free behavioral patterns. We find that, under certain carefully-chosen parameterizations, the classic patterns arising side by side, with policy *π* displaying the model-based profile, and *π*_0_ the model-free pattern (Fig 4C). Because *π* dictates the overt behavior of the agent, the latter displays a model-based pattern, as also seen in human performance in some studies [59]. When *RNN*_*π*_ is ablated, behavior then shifts away from the model-based pattern, in line with the observation that disruption of prefrontal function decreases model-based control in the two-step task [60, 61].

This differentiation of function arises, as in the previous simulations, from the MDL-C optimization objective. As has been noted in the literature on model-based versus model-free learning, the latter is less algorithmically complex [9]. The simplicity bias in MDL-C, imposed on *π*_0_, therefore tilts that policy toward the actions that would be chosen by a model-free agent. Policy *π*, meanwhile, can reap a bit more reward by implementing a policy that takes task structure more fully into account.

Work with the two-step task has consistently found that both humans and animals show a variety of “mixed” patterns [50, 63, 64] distinct from either of the classic patterns. It has also cast doubt on the idea that these patterns, quantified from behavior, map 1:1 onto other measures of goal-directed or habitual control [59, 62, 65]. When we train MDL-C over a broader range of hyperparameters (see S1 Methods), we observe similar mixed patterns across large portions of the parameter space (Figs 4D and B-D in S1 Appendix), and that either primarily “model-based”, “model-free” or “perseverative” behavior can appear in either *π* or *π*_0_. Thus, while a clean separation between model-based and model-free learning can arise within MDL-C, such a division is not hardwired into the framework. Depending on the precise setting, minimizing the description length of behavior can also lead to graded intermediate patterns, providing leverage on some otherwise problematic experimental observations [62].

**Fig 5:**
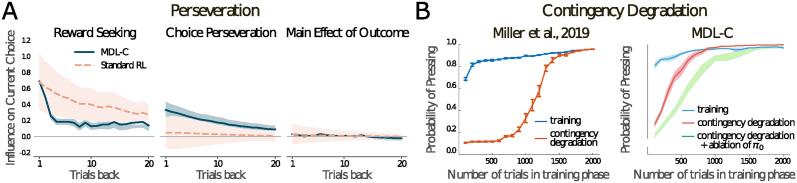
**A**. Logistic regression weights showing the influence on the current action of reward contingent on choice (reward seeking), previous choices (perseveration), and reward independent of choice (main effect of outcome) of MDL-C and a standard RL agent on the drifting two-armed bandit task from [57]. MDL-C displays a stronger tendency towards perseveration, reminiscent of rats trained on the same task. **B**. Left: Simulation of contingency degradation from [14]. The longer the training phase (x axis), the longer lever-pressing persists after reward is discontinued (red). Right: Corresponding behavior from MDL-C, also showing the effect of ablating *π*_0_.

While the two-step task has been an important driver of dual-process theory in the domain of reward-based learning, important insights have also come from studies of instrumental learning. One key feature of animal behavior within this domain is *perseveration*: the tendency to repeat previous actions independent of their association with reward. [57] administered a two-arm bandit task to rats, where the probability of one of two ports delivering a juice reward drifted randomly across trials. Performing logistic regression on different features of the last 20 trials showed that past choices contingent on reward and the repetition of previous actions had a strong influence on behavior on the current trial. We simulated this experiment, and found that agents trained for simple reward maximization were influenced by previous rewards contingent on choices, but did not display perseverative tendencies, while MDL-C agents exhibited both (Fig 5A, details in S1 Methods).

Another important experimental manipulation within this literature is known as *contingency degradation*. Here, rewards are at first delivered only in response to a particular action, but then later are delivered in a non-contingent manner, independent of whether the action was selected. Unsurprisingly, this change typically triggers a shift away from the action in question. Critically, however, this adjustment is reduced or slowed if the initial training with reward was extensive [9, 14, 66] (Fig 5B). Prevailing explanations for this effect share a dual-process perspective, according to which insensitivity to contingency degradation reflects a transfer of control from one learning process that is relatively flexible to another which adjusts less quickly [9, 14]. Consistent with this account, lesions to dorsolateral striatum—a structure proposed to be involved in that latter system—partially protects against training-induced inflexibility [67].

MDL-C captures the empirically observed effects of contingency degradation, but also offers a novel computational perspective. As shown in Fig 5B, the speed with which the MDL-C agent reduces its response rate after contingency degradation depends on how long the agent was previously trained with reward (see S1 Methods for simulation details). As in the experimental data, behavior becomes less flexible as the duration of training increases. This shift is a result of the MDL-C optimization objective. Policy *π* is initially able to adjust rapidly, responding to reward by emitting the rewarded action frequently. If contingency degradation occurs immediately, *π* is able to adapt flexibly. However, if reward continues for a longer period, the rewarded policy gradually comes to be mirrored in *π*_0_, driven by the third term in Eq 2. Once *π*_0_ becomes strongly biased toward the rewarded action, it is difficult for policy *π* to diverge from this pattern, again due to the third term in Eq 2 (an effect that is attenuated if *π*_0_ is ablated, analogous to lesioning dorsolateral striatum; see Fig 5B). This computational mechanism is related to others that have been proposed in models devised specifically to account for contingency degradation effects, based on uncertainty or habit strength [9, 14] (see S1 Appendix). However, MDL-C ties the relevant learning dynamics to a higher-level computational objective, namely, minimizing the description length of behavior (compare [23, 68]).

#### Simulation 3: Judgment and decision making

As noted earlier, dual-process models in JDM research distinguish between System-1 and System-2 strategies, the former implementing imprecise heuristic procedures, and the latter sounder but more computationally expensive analysis [6, 7]. As in the other dual-process domains we have considered, there appears to be a neuroanatomical dissociation in this case as well, with System-2 responses depending on prefrontal computations [15, 16].

**Fig 6:**
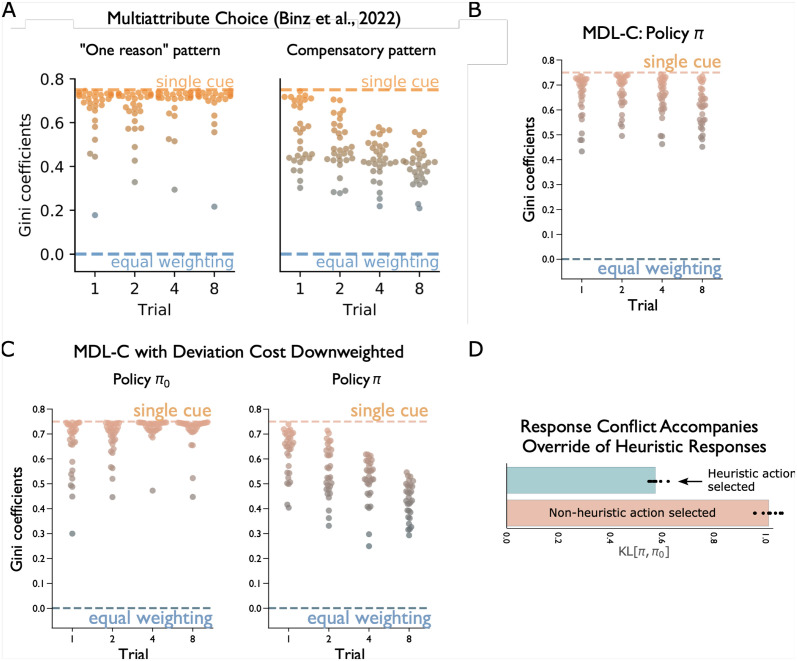
**A**. Heuristic one-reason decision making (left) and richer compensatory decision making (right) in a multi-attribute choice task, from [24]. Gini coefficients, on the y axis, capture the degree to which decisions depend on one feature (higher values, with asymptotic maximum of one) versus all features evenly (zero), with references for one-reason decision making (*single cue*) and a fully compensatory strategy (*equal weighting*) indicated. Data points for each trial correspond to observations from separate simulation runs. Human participants in the study displayed both patterns of behavior, depending on the task conditions. **B**. Behavior of MDL-C in the task from [24], under conditions where human participants displayed one-reason decision making. **C**. Behavior of *π*_0_ (left) and *π* (right) when the KL penalty for divergence between the two policies is reduced (see S1 Methods). **D**. In the simulation from panel C, the divergence between policies is increased when the agent emits a non-heuristic decision.

Recent research on heuristics has increasingly focused on the hypothesis that they represent resource-rational approximations to rational choice [26]. In one especially relevant study, [24] proposed that heuristic decision making arises from a process that “controls for how many bits are required to implement the emerging decision-making algorithm” (p. 8). This obviously comes close to the motivations behind MDL-C. Indeed, [24] implement their theory in the form of a recurrent neural network, employing the same regularization that we apply to our $RNN_{\pi_{0}}$. They then proceed to show how the resulting model can account for heuristic use across several decision-making contexts. One heuristic they focus on, called *one-reason decision making*, involves focusing on a single choice attribute to the exclusion of others [69]. As shown in Fig 6A, reproduced from [24], a description-length regularized network, trained under conditions where one-reason decision making is adaptive (see [24] and S1 Methods), shows use of this heuristic in its behavior, as also seen in human participants performing the same task. In contrast, an unregularized version of the same network implements a more accurate but also more expensive “compensatory” strategy, weighing choice features more evenly.

As illustrated in Fig 6B, when MDL-C is trained on the same task as the one used by [24] (see S1 Methods), it displays precisely the same heuristic behavior those authors observed in their human experimental participants.

Digging deeper, MDL-C provides an explanation for some additional empirical phenomena that are not addressed by [24] or, to the best of our knowledge, any other previous computational model. In an experimental study of one-reason decision making, [69] observed that application of the heuristic varied depending on the available payoffs. Specifically, heuristic use declined with the relative cost of applying a compensatory strategy, taking more feature values into account. MDL-C shows the same effect. When the weighting of the deviation term *D*_*KL*_(*π*||*π*_0_) is reduced relative to the value-maximization term in the MDL-C objective (see S1 Methods), the policy *π* and thus the agent’s behavior take on a non-heuristic compensatory form (Fig 6D). Critically, in this case MDL-C instantiates the non-heuristic policy side-by-side with the heuristic policy, which continues to appear at the level of *π*_0_. This aligns with work suggesting that System-1 decision making can occur covertly even in cases where overt responding reflects a System-2 strategy. In particular, [15] observed activation in prefrontal areas associated with conflict detection in circumstances where a tempting heuristic response was successfully overridden by fuller reasoning (see also [16]). A parallel effect is seen in our MDL-C agent in the degree of conflict (KL divergence) between policies *π* and *π*_0_ (Fig 6D).

#### Comparison with previous models

To our knowledge, no previous computational model has simultaneously captured the core dual-process phenomena we’ve considered, thereby bridging the domains of executive function, reward-based decision making and JDM. However, a range of previous models have addressed the relevant phenomena in a fashion limited to one of those domains. Having stressed the unifying, cross-disciplinary character of the present work, it is also befitting to consider the relationships between MDL-C and these domain-specific models. Particularly important is the question of whether such domain-specific models capture any empirical phenomena that MDL-C might have difficulty addressing.

**Fig 7:**
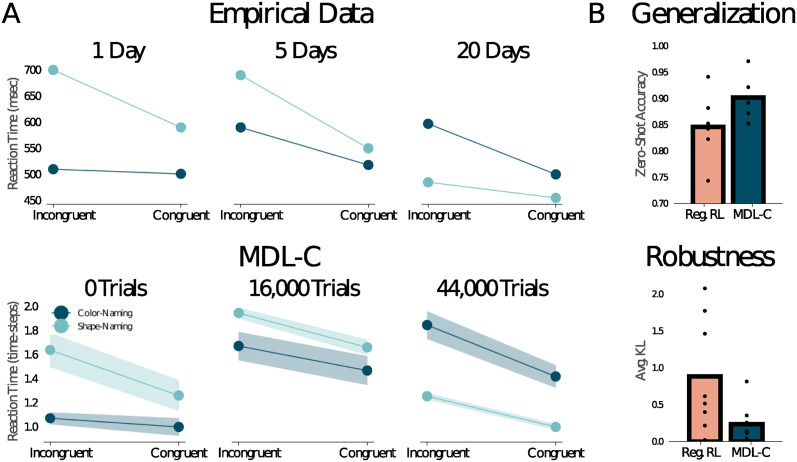
**A**. Top: Behavioral data from the modified Stroop task studied by [71]. Early in training, shape-naming responses were both slower than color-naming responses and more affected by stimulus congruence, consistent with shape-naming being the relatively “controlled” response and color-naming relatively “automatic.” With extensive training, the pattern flipped, with shape-naming becoming faster than color-naming and less affected by stimulus congruence. Bottom: Under training conditions mimicking the experimental study, MDL-C displayed a similar pattern of behavior, with a significant main effect of task and a significant interaction between task and trial-type (*p* < 0.05) at both 0 trials and 44,000 trials. **B**. Zero-shot Stroop performance in MDL-C and an unregularized baseline model (see S1 Methods). Top: Color-naming accuracy on incongruent Stroop stimuli, after training only with neutral stimuli (see main text and S1 Methods). Bottom: KL divergence between action probability distributions under two conditions, (1) presentation of incongruent Stroop stimuli, and (2) presentation of Stroop stimuli with the word identity input masked out. MDL-C shows significantly lower divergence, indicating that the control policy attends less to the task-irrelevant factor—i.e., MDL-C is more robust to distractors—despite never having been trained on incongruent stimuli.

In the area of executive control, our model bears strong connections with the classic connectionist model proposed by [17]. In particular, both characterize the distinction between controlled and automatic processing as arising from learning. To illustrate this point, Cohen and colleagues [70] modeled results from a behavioral study by [71] (Fig 7A). Here, participants were presented with colored shapes, and asked either to name their color or to announce a color name that had been arbitrarily assigned to the relevant shape (e.g., a particular irregular pentagon might be given the name *blue*, independent of its display color). Interference between the two tasks was quantified by comparing response time on incongruent trials, where color- and shape-name conflicted, against congruent trials, where they matched. Early in training, interference was larger for the shape-naming task than the color-naming task, suggesting that color-naming was relatively “automatic” and shape-naming relatively “controlled.” However, after extensive training on the shape-naming task the pattern flipped, consistent with the idea that within-task learning had rendered shape-naming relatively “automatic.” This effect was well captured by the neural network model of [70], and it also arises in our MDL-C model (see Fig 7A and S1 Methods).

As this example illustrates, gradual learning processes, operating over the course of extensive practice on relevant tasks, are important to the theoretical account we are proposing with MDL-C. On the face of it, this may seem to stand in tension with how learning occurs in most human behavioral experiments, where participants dive in on novel tasks given little more than some verbal instructions and few practice trials. For example, in the classic Stroop task, it seems reasonable to assume that participants have rarely if ever been asked to name the color of a word that itself names a color, but they do this “zero-shot,” and immediately display Stroop interference. To show that our MDL-C implementation accommodates this kind of zero-shot learning, we trained our agent on color-naming and on word-reading, only ever presenting “neutral” stimuli, omitting the word feature during color-naming and omitting the color feature during word-reading (see S1 Methods). At test, incongruent feature sets were presented. The model responded correctly on the vast majority of trials given the task-cue input—performing significantly better than an ablated network lacking MDL regularization—but also showed Stroop interference (see Fig 7b). In recent work, [72] have shown how neural networks can follow verbal instructions zero-shot in a wider range of tasks. It would be exciting to expand our MDL-C implementation to incorporate greater behavioral breadth and flexibility in the same way.

Elaborations of the [17] model have offered a mechanistic explanation for the special role played by prefrontal cortex in representing aspects of context, attributing to prefrontal circuits a special set of gating-based memory mechanisms [73]. MDL-C offers a complementary account, instead addressing why it makes sense in normative terms for the brain to support both control and habit systems (see [22] for a related but domain-specific analysis). It is important to emphasize, however, that we are not attempting to claim that MDL-C’s *RNN*_*π*_ and $RNN_{\pi_{0}}$ map directly onto specific brain regions, but rather only that the split architecture of our MDL-C agents reflects evidence supporting neuroanatomical divisions between areas of controlled and automatic processing. As it turns out, however, MDL-C does in fact give rise to a solution that gates different information into different parts of the information-processing architecture, broadly consistent with gating-based models of cognitive control [73]. From the point of view of our theory, such gating mechanisms might be viewed as solutions to the MDL-C problem discovered by evolution rather than online learning. It is worth noting that some of the most recent work to apply the notion of gating to PFC function has postulated a multilevel hierarchy, deeper than the one we consider in our simulations. There is no practical impediment to extending the MDL-C architecture to include multiple hierarchical levels; a natural approach would be to regularize each pair of adjacent layers with respect to one another, varying the weight of the complexity cost monotonically across layers. We have not, however, implemented this idea and it therefore stands as an appealing opportunity for next-step research. Another elaboration of the [17] model adds a “cost of control,” a negative utility attached to the overriding of default response-selection processes [10, 25, 49, 74]. As noted in our simulation of demand avoidance, the deviation term in the MDL-C objective effectively imposes a cost of control, showing how this cost fits into a broader optimization process. While philosophically aligned, MDL-C differs from these models in important ways, most significantly in that its default policy is *learned*. That is, while the control policy may be learned using a similar objective (e.g., [25] also use KL-regularized policy optimization with respect to a default policy), MDL-C directly models the acquisition of automatic/habit-like behavior as the minimization of an MDL-based objective, whereas most previous sequential decision-making approaches modeling a cost of control do so with respect to a fixed default policy.

The classic [17] model has been elaborated in subsequent work to address another canonical phenomenon in the executive function literature, which we have not previously touched upon: task-switching costs (see, e.g., [75–77]). Importantly, in order to capture switch-cost effects, including such phenomena as residual and asymmetric switch costs, the relevant computational models have had to build in temporally and mechanistically fine-grained accounts of working memory function, modeling attractor dynamics and hysteresis effects that fall well below the level of abstraction our MDL-C implementation occupies. It would be informative to implement MDL-C with an increased level of temporal granularity (as for example in [75]) and to evaluate task-switching effects in this setting.

We turn now from executive function to reward-based decision making. As shown in Simulation 2, when MDL-C operates within an appropriate task context, it can yield side-by-side decision mechanisms with profiles matching model-based and model-free control. This links MDL-C with a wide range of recent models of reward-based decision making, which center on this side-by-side configuration [4, 9, 52, 53]. As discussed under Results, the empirical data motivating those dual-system models is complex. In particular, neural activity aligning with model-free computations is not always “pure” of model-based characteristics (see, e.g., [50]). Such computational purity is not enforced in MDL-C, either, and under some parameterizations MDL-C displays the same intermediate patterns that have been observed in some experimental studies. (Indeed, such mixed patterns were seen across most of the parameter space we explored; see Figs B-D in S1 Appendix). The interpretation of ostensibly model-based behavior in the two-step task is also nuanced [51, 58]. However, we have demonstrated elsewhere [78] that genuinely model-based computations can arise within recurrent neural networks under conditions comparable to those employed in the present work.

Beyond model-based and model-free RL, the dynamics of habit acquisition in MDL-C also link it with recent models that replace model-free RL with a reward-independent, practice-based learning mechanism [14, 79, 80]. The learning mechanism of MDL-C’s default policy is closely related to these, with two important differences. The first is that the practice-based learning mechanisms adopt as the target of learning the discrete actions actually taken by the agent, while MDL-C’s default policy adopts as its target the full probabilistic control policy from which those actions are sampled. The second is that the addition of VDO effectively regulates the complexity of the habits that can be learned and the rate at which habit formation occurs. The results presented in Fig 5G support this connection. Of particular interest, a recent study provided evidence that dopamine dynamics in a posterior sector of the striatum encode not a reward-prediction error, but instead an *action*-prediction error, which drives situation-action associations [81]. This aligns quite closely with how learning operates in $RNN_{\pi_{0}}$ in our MDL-C implementation, where weight updates are driven by a mismatch between the actions predicted by *π*_0_ and those dictated by *π*.

Practice-based accounts of habits have been proposed [14] to explain not only classic assays of habits, but also trial-by-trial perseveration, an effect in which subjects tend to repeat in the future choices that have been made in the past, regardless of the associated stimuli and outcomes [57, 82–84]. To test whether MDL-C would show such effects, we ran it on a drifting two-armed bandit task, in which rats show robust perseveration [57]. We find that MDL-C shows similar perseveration, while an ablation model lacking the default policy does not (Fig 5F).

Despite all of these connections, MDL-C differs from most previous models in that it does not involve a direct competition between control systems [9, 85]. In MDL-C, the policy *π* always has the last word on action selection, which may be to either endorse or override default policy *π*_0_ (as discussed above). Interestingly, this relationship between systems resembles one proposal for the interplay between System 1 and System 2 in the JDM literature, according to which “System 1 quickly proposes intuitive answers to judgment problems as they arise, and System 2 monitors the quality of these proposals, which it may endorse, correct or override” [86].

Within the JDM literature, among computational models of heuristic judgment, our account aligns closely with the one recently proposed by [24], adding to it in the ways noted earlier. Like [24], we have only applied MDL-C to a small set of heuristics from among the many considered in the JDM literature. An important challenge, both for MDL-C and for the [24] account, will be to test applicability to a wider range of the relevant behavioral phenomena. Needless to say, a still wider range of decision effects addressed by the JDM literature, from risk attitudes to self-control conflicts, remain untouched by the present introductory work, and the compatibility of the our theory with such effects will necessarily await further research.

Some readers will have remarked that the our account of dual-process control shares important characteristics with a range of research on “resource-rational” cognition [26], where limitations on computational capacity are understood to constrain strategies for adaptive information processing. In the context of goal pursuit, this perspective has given rise to the notion of a value-complexity tradeoff, where reward maximization balances against the cost of encoding or computing behavioral policies [23, 24, 87, 88]. While our computational account resonates strongly with this set of ideas, two qualifying points call for consideration. First, a great deal depends on the exact nature of the computational bottleneck hypothesized. At the center of our account is a measure related to algorithmic complexity [24, 30, 33], a measure that differs from the mutual information constraint that has provided the usual focus for value-complexity tradeoff theories [23, 89] (see S1 Methods). Second and still more important, the MDL-C framework does not anchor on the assumption of fixed and insuperable resource restrictions. The relevant limitations on complexity are regarded not as inherent to neural computation, but rather as advantageous for representation learning and generalization [90]. Indeed, while reward-complexity tradeoff models typically involve a single bottlenecked processing pathway [23, 24], MDL-C includes a second pathway that allows the agent to work around constraints on computational capacity. This allows for the formation of expressive, task-specific representations alongside more compressed representations that capture shared structure across tasks [22].

### Discussion

Dual-process structure appears ubiquitously across multiple domains of human decision making. Though this is almost certainly a simplification and action selection lies along a spectrum from controlled to automatic, this tradeoff has been a useful axis for studying behavior. While this has long been recognized by psychological and neuroscientific models, only recently has the normative question been raised: Can dual-process control be understood as solving some fundamental computational problem? MDL-C, an approach for efficient multitask RL from the machine learning literature, can be derived directly from the demands of generalization and sequential decision-making, without reference to neuroscientific data. Despite this independent theoretical lineage, MDL-C turns out to provide a compelling explanation for dual-process structure.

The account we have presented is also distinctive for its unifying character. Although sophisticated dual-process models have been proposed within each of the behavioral domains we have considered in the present work—executive control (e.g., [74]), reward-based decision making (e.g., [9]), and JDM (e.g., [24])—to our knowledge MDL-C is the first computational proposal to account for empirical phenomena across all three of these fields. However, our treatment of the neuroscientific issues has, of necessity, been quite broad; important next steps for developing the theory would, for example, be to provide a more detailed account of MDL-C’s relationship with specific neuroanatomical structures, particularly regional distinctions and hierarchical organization within prefrontal cortex [91]. While we view MDL-C as a promising step in the direction of providing unified account of dual process phenomena across fields, deep questions remain and further work needs to be done.

Beyond psychology and neuroscience, MDL-C, with its origin in machine learning [38], bears a number of important links with existing work in that field. In particular, it belongs to a broad class of RL systems that employ regularized policy optimization, where the agent policy is regularized toward some reference or default (see [92]). Most relevant are approaches where the default policy is itself learned from experience [93–96]. In previous work involving such learning, it has been deemed necessary to stipulate an ‘information asymmetry,’ imposing some hand-crafted difference between the observations available to the control and default policies [25, 93–95]. MDL-C allows this information asymmetry itself to be learned, as our simulations have demonstrated (see Figs 2C, 3B, 5E and 6D). Given this point and others, we are hopeful that further insights gained into MDL-C’s relationship with biological cognition could spur modifications that provide benefits in a machine learning context as well.

## Supporting information

S1 Appendix Additional discussion of our approach and supplemental figures. (PDF)

S1 Methods Detailed descriptions of the discussed simulations. (PDF)
