# Supplementary material for: Understanding dual process cognition via the minimum description length principle
Source: PLoS Comput Biol. 2024 Oct 18;20(10):e1012383. doi: 10.1371/journal.pcbi.1012383 (PMC11534269; doi:10.1371/journal.pcbi.1012383)
Supplement: S1 Appendix — (PDF) [file pcbi.1012383.s001.pdf]

Supporting information — supplementary discussion

Computational Framework

We discuss here several details of the MDL-C computational framework.

In importing MDL into reinforcement learning, we made a link between the 'data' to be compressed and the agent's policy. Several additional comments are warranted concerning this choice. First, it is important to note that MDL, or compression more generally, can also be applied quite naturally to other data structures within RL. One obvious target for compression is the action-outcome model that lies at the center of model-based reinforcement learning, and this is just one of several candidates (see [115]). While our MDL-C proposal focuses on the agent policy, this in no way excludes other compression targets.

Directing MDL toward the agent policy results in a setup that differs in some subtle and interesting ways from what is involved in classical MDL. In the latter setting, the data are typically assumed to be fixed. In MDL-C, in contrast, the data (since they comprise the agent's policy) are subject to continual change. Furthermore, while in classical MDL the data are assumed to be independent of MDL itself, the data in MDL-C can be altered over time in response to pressures that arise from the MDL objective. This feature could be avoided if MDL-C were implemented as a strict constrained optimization process, with no trade-off between reward maximization and compression. However, as it turns out, some of the empirical phenomena addressed in the main paper arise specifically from the trade-off that our implementation involves. One obvious example of this is the demand-avoidance effect described in Simulation 1, where the policy is clearly influenced by the compression terms in the MDL-C objective. An interesting target for next-step research would be to consider the possible psychological and neuroscientific implications of this hypothesized trade-off, and in particular to consider whether a clear normative justification for this feature of MDL-C might be identified.

As discussed under Methods, the trade-off between value and compression in MDL-C is controlled by an adjustable hyperparameter. As also noted there, our neural network implementation also includes hyperparameters weighting the complexity and deviation terms against one another. This may seem surprising to readers familiar with classical MDL, where no such relative weighting occurs. However, it should be noted that the complexity term in classical MDL directly quantifies algorithmic or Kolmogorov complexity (see [38]), whereas our implementation quantifies complexity in terms of the weight distribution of a neural network. This weight distribution serves as a proxy for algorithmic complexity, since it affects the complexity of the policies the network implements. However, it is not identical, nor is it guaranteed to quantify complexity on a similar scale, thus requiring the introduction of a scaling parameter. A similar point pertains to the other KL cost in the objective function used in our implementation, capturing the divergence between policies π and π_0. This, too, is a proxy for the corresponding term in classical MDL, which again is intended to capture algorithmic complexity.

An additional aspect of our MDL-C implementation that bears further discussion is the process by which RNN_π interfaces with RNN_{π_0} at decision or inference time. In our neural network implementation, at least as the code is written, the interaction is quite straightforward: RNN_{π_0} outputs its policy and then this is simply overwritten by RNN_π. This way of describing the interaction may appear to stand in tension with our description in the main text of RNN_π "overriding" or "endorsing" RNN_{π_0}. However it should be noted that there is a notational variant of our implementation that aligns much better with these descriptions. Specifically, one can view RNN_π as *adding* or *subtracting* from the action probabilities specified by RNN_{π_0} (or, alternatively,

adjusting them in a multiplicative fashion), with the result corresponding to π. If RNN_π is viewed as outputting a vector of differences or deltas, then an output of zero can be interpreted as an "endorsement" of RNN_{π_0}, and any other output can be interpreted as "overriding" RNN_{π_0}. We emphasize here that this is simply a different interpretation of the same process at inference time, not an alternative training regime.

One final comment on the computational framework relates to the claims in the main text about generalization performance. These may appear to stand in tension with some of the phenomena simulated in our experiments. For example, the behavioral inflexibility seen in contingency degradation after extended pre-training may appear to contradict the idea that compression, in the style of MDL-C, fosters rapid adaptation to new task challenges. However, it should be noted that the idea of 'generalization' can cut both ways. Adversarial environments can be constructed where an agent's tendency to base action selection on past outcomes yields what looks like maladaptive behavior. Contingency degradation with extensive pretraining can be seen as adversarial in this sense. The claim that dual-process organization supports generalization on average is thus reconcilable with cases where it can be understood to cause locally suboptimal behavior.

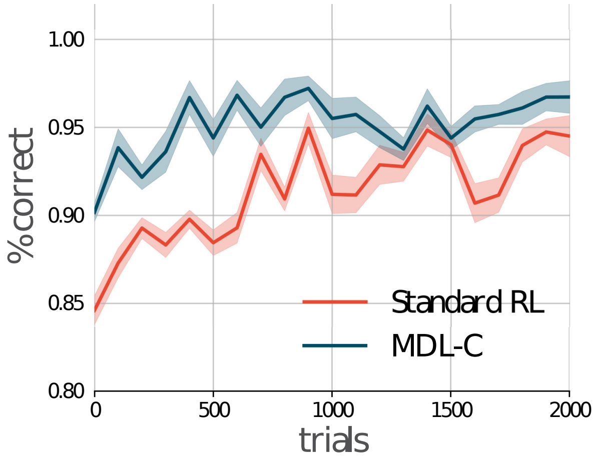

Fig A Pre-training with MDL-C enables agents to better ignore distracting features during fine-tuning.

In the main text, we motivate MDL-C by focusing on the problem of generalization. It may seem surprising, then, that none of the dual-process phenomena addressed in our Results section involve behavioral generalization. This is, of course, not a flaw in our account. Our simulations show that dual-process phenomena can be understood as reflecting the operation of a mechanism that *elsewhere and more generally* supports behavioral adaptation. This point is illustrated by juxtaposing the results presented in Fig 2 in the main text from those presented in Fig 1B (left), since both relate to navigation. As a stimulus to further research, it is worth describing an informal, exploratory simulation in which we investigated the role that generalization might be understood to play in one other task we addressed in our simulations, namely the Stroop task. The Stroop task can be understood as reflecting a simple form of flexible generalization: People performing the task are able, based on a verbal instruction, to ignore word identity and name colors, despite never (or at least rarely) having encountered colored *color words* in a color-naming task context before. To study this kind of flexibility in MDL-C, we trained our network agent to perform color-naming on inputs indicating color but not word identity, and also to perform word-reading on inputs indicating color-word identity but not color *per se*. As soon as training had

proceeded far enough to yield error-free task performance, we introduced Stroop inputs including both color and color-word information. Given a task cue, the agent responded much more accurately to such inputs than a baseline agent trained without description-length regularization, that is, using only the RL term in the MDL-C objective (Fig A). This simple result suggests that MDL-C learned to 'attend' only to task-relevant input channels during the initial training, preparing it to attend selectively when faced with Stroop stimuli. It is our hope and expectation that further simulation work along lines such as these may generate further testable predictions from MDL-C in the task settings addressed in our simulations of dual-task phenomena.

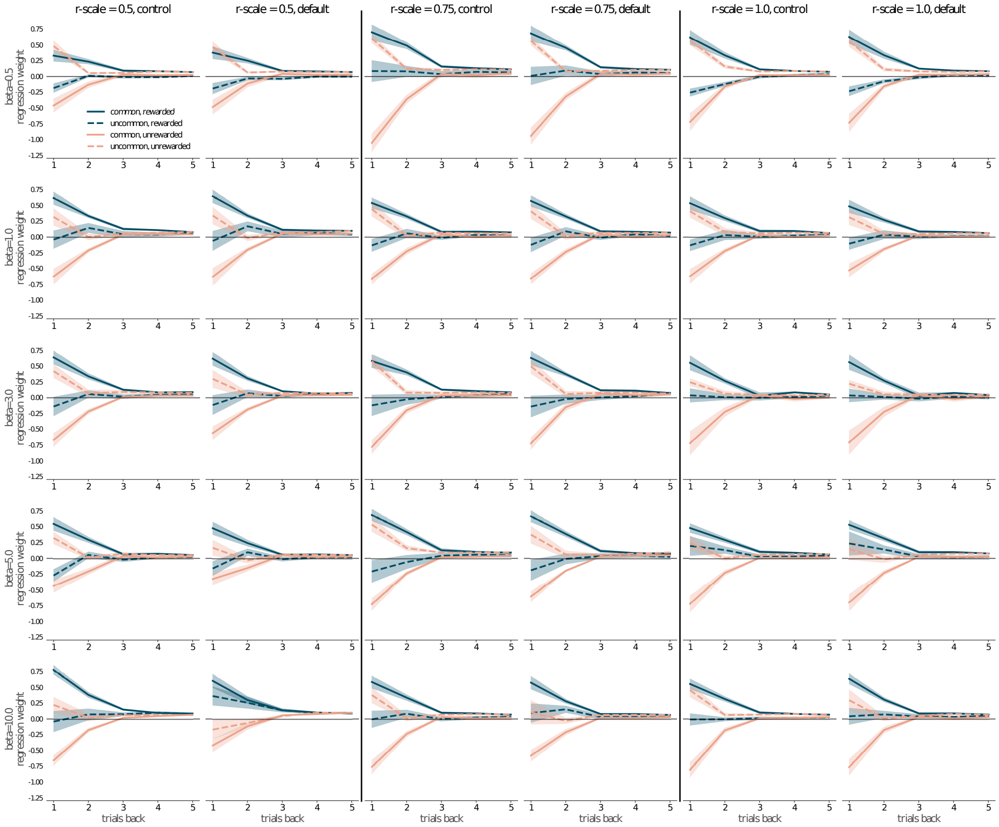

Fig B Two-step results from full hyperparameter sweep described in Methods, with $\alpha = 0.05$. Format as in Fig 5 in the main text.

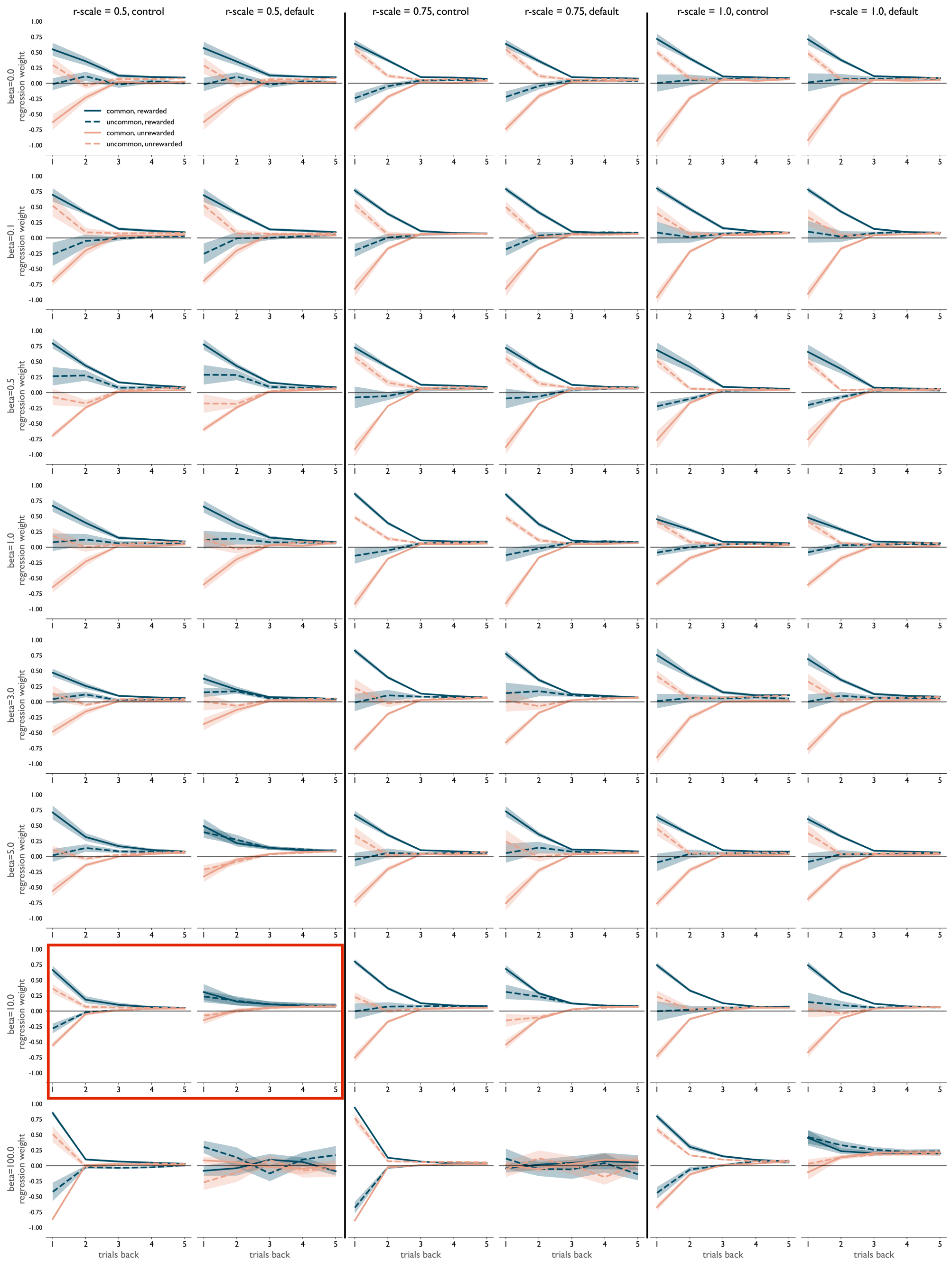

Fig C Two-step results from full hyperparameter sweep described in Methods, with $\alpha = 0.1$. The boxed plot appears in Fig 5D in the main text. Format as in Fig 5 in the main text.

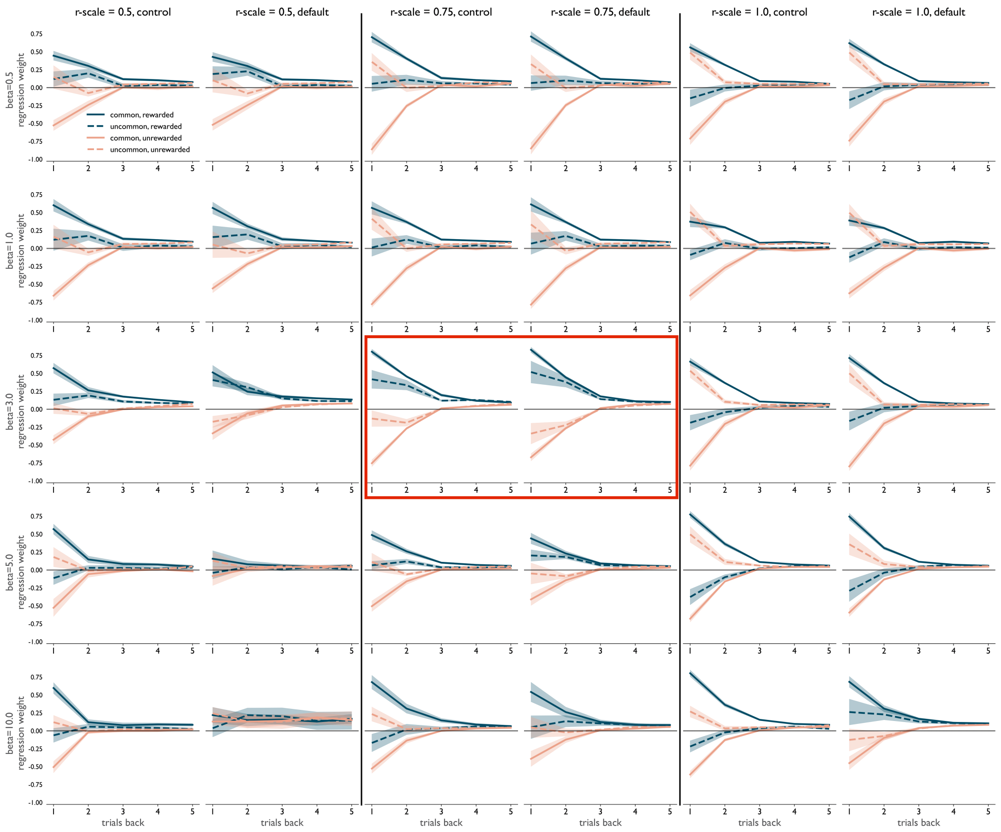

Fig D Two-step results from full hyperparameter sweep described in Methods, with $\alpha = 0.2$. The boxed plot appears in Fig 5E in the main text. Format as in Fig 5 in the main text.
