# Supplementary material for: Understanding dual process cognition via the minimum description length principle
Source: PLoS Comput Biol. 2024 Oct 18;20(10):e1012383. doi: 10.1371/journal.pcbi.1012383 (PMC11534269; doi:10.1371/journal.pcbi.1012383)
Supplement: S1 Methods — (PDF) [file pcbi.1012383.s002.pdf]

Supporting information — online methods

Architecture and learning algorithm

All experiments employed a common architecture and learning algorithm with minor implementational variations across simulations. Our implementation of MDL-C consists of two recurrent neural networks (RNNs) which we call the *control policy network* RNN_π and the *default policy network* RNN_{π_0}. These RNNs have identical sizes and architectures. They are also provided with identical inputs for each time step of experience, consisting of a one-hot encoding of the previous action, a scalar indicating the previous reward, and a vector of task-specific information (the "observation") which will be described separately for each task.

The control policy network RNN_π produces as output a vector of *policy* logits π which determine the probability of taking each available action, as well as a scalar *value* estimate of its expected future reward from the current state. It is trained using the *advantage actor-critic* (A2C; [83]) algorithm, which encourages it to produces actions which maximize expected long-term reward $\mathbb{E}_\pi[R]$. The control policy network is also regularized using a term which encourages its action probabilities to match those produced by the default policy network. This is equivalent to encouraging the conditional description length $L(\pi|\pi_0)$ to be low.

The default policy network also produces as output a vector of policy logits π_0. In the intact system, these are overwritten by the control policy network (see Supplementary Discussion), and so serve primarily to regularize the control policy. The default policy network is trained by *policy distillation* [84,107] to match the output of the control policy network π. It is regularized using *variational dropout* (VDO; [85]), which encourages its absolute description length $L(\pi_0)$ to be low.

The overall MDL-C objective for π and π_0 can be written

$$\mathcal{L}_{\mathrm{MDL-C}} = \mathbb{E}_\pi[R] - [\alpha D_{KL}(\pi(a|x_t;\theta)||\pi_0(a|x_t;w)) + \beta \bar{D}_{KL}(q(w;\phi)||p(w))],$$

corresponding to the reward maximization, complexity, and goodness-of-fit terms introduced in Equation (2). Note that this expression introduces an additional weighting parameter relative to Equation (2), the rationale for which is presented in our Supplementary Discussion. Also, as will become clear in what follows, the overall objective above can be decomposed and sub-parts used to train different sectors of our agent, since only certain terms affect different pathways. Below, we describe each term in the objective in detail.

Control policy network Unless otherwise noted, the control policy RNN_π was trained via a modification of Advantage Actor-Critic (A2C), which is described in detail in [83,86]. Briefly, A2C is an on-policy actor-critic algorithm which weights gradients by a Monte-Carlo estimate of the advantage at each time step. In order to prevent premature convergence to suboptimal local maxima, [83] add an entropy bonus to the objective to prevent the policy from becoming overly deterministic early in training. In MDL-C, entropy regularization is replaced with a Kullback-Leibler (KL) divergence

penalty with respect to the default policy distribution π_0:

$$\begin{aligned}
\nabla\mathcal{L}_\pi &= \nabla\mathcal{L}_{A2C} + \alpha\nabla\mathcal{L}_{KL}, \quad \text{where} \\
\mathcal{L}_{A2C} &= -\delta_t(x_t;\theta_v)\log\pi(a_t|x_t;\theta) + \frac{\alpha_v}{2}\delta_t(x_t;\theta_v)^2, \\
\mathcal{L}_{KL} &= D_{KL}(\pi(a|x_t;\theta)||\pi_0(a|x_t;w)), \\
\delta_t(x_t;\theta_v) &= R_t - v(x_t;\theta_v), \\
R_t &= \sum_{i=1}^{k-1}\gamma^i r_{t+i} + \gamma^k v(s_{t+k};\theta_v),
\end{aligned}$$

where $x_t = [s_t, a_{t-1}, r_{t-1}]^\top$ is the observation vector at time t consisting of the state s_t, previous action a_{t-1}, and previous reward r_{t-1}, θ are the control policy parameters, α_v is a hyperparameter controlling the weight on the value-learning loss, θ_v are the value function parameters, $D_{KL}(q||p) = \sum_a q(a)\log\frac{q(a)}{p(a)}$ is the KL divergence between distributions q and p, w are the sampled parameters of the default policy network (details below), and γ is a scalar discount factor. This KL-regularized RL framework has a rich theoretical and experimental history in both machine learning and neuroscience [79, 135], and can be derived (depending on the direction of the KL cost) through an interpretation of RL as Bayesian inference [136]. Moreover, it has been shown that this approach guarantees accelerated convergence compared to non-regularized methods under the condition that the tasks faced by the agent induce optimal policies which behave similarly [80]. Intuitively, the control policy network is trained to maximize reward while simultaneously being encouraged to remain close to the behavior encoded by the default policy π_0. Early in training, obtaining reward may be challenging, and so the control policy network is primarily taught via learning signals generated by the default policy network. If the default policy network encodes behavior that is useful for the task, then learning from it may enable the control policy network to obtain reward earlier, accelerating training. In multitask settings where π_0 is conserved across tasks, it is therefore important that π_0 encodes behavior which is generally useful for the tasks faced by the agent. For further detail on KL-regularized multitask policy optimization, we refer readers to [80, 112].

Default policy network The default policy was trained off-policy via distillation [84] from the control policy network (in other words, the default policy aims to match the control policy distribution) offset by a regularization penalty on the effective bit length encoding of the network parameters:

$$\begin{aligned}
\nabla\mathcal{L}_{\pi_0} &= \nabla\mathcal{L}_{\text{distill}} + \nabla\mathcal{L}_{\text{VDO}} \\
&= \sum_{k=1}^{M}\nabla_\phi D_{KL}(\pi(a|x_k;\theta)||\pi_0(a|x_k;w=f(\phi;\epsilon))) + \beta\nabla_\phi\bar{D}_{KL}(q(w;\phi)||p(w)); \\
f(\phi^{(i)};\epsilon) &= \phi_0^{(i)}(1+\sqrt{\phi_\alpha^{(i)}}\epsilon^{(i)}); \quad \epsilon^{(i)} \sim \mathcal{N}(0,1),
\end{aligned}$$

where M is the minibatch size of data sampled from an experience replay buffer [106], w are default policy parameters sampled using the reparameterization trick [85] to allow for automatic differentiation, β is a scalar hyperparameter weighting the regularization, and $\phi = \{\phi_0, \phi_\alpha\}$ are learned parameters defining the distribution over default policy parameters: $q(w;\phi) = \prod_i \mathcal{N}\left(w^{(i)};\phi_0^{(i)},\phi_\alpha^{(i)}(\phi_0^{(i)})^2\right)$, where the superscript (i) denotes the ith parameter, and $p(w)$ is the log-uniform prior $p(|w^{(i)}|) \propto 1/|w^{(i)}|$. The noise ϵ—and therefore, effectively, the default policy weights w—are re-sampled after every

episode of training. It's this particular form of noise which limits the effective capacity of RNN_{π_0}. We use the average KL,

$$\bar{D}_{KL}(q(w;\phi)||p(w)) = \frac{1}{N}\sum_{i=1}^{N} D_{KL}(q(w^{(i)};\phi^{(i)})||p(w^{(i)})).$$

The regularization loss is computed and minimized using *variational dropout* (VDO; [42,85]), which uses a local reparameterizaztion trick to implement this KL regularization as a particular form of multiplicative noise placed on the network weights. Regularizing with respect to this choice of prior has the effect of limiting the effective bit-length of the parameters of RNN_{π_0}, reducing its effective complexity [85]. Note that the distillation loss $D_{KL}(\pi(a|x_k;\theta)||\pi_0(a|x_k;w=f(\phi;\epsilon)))$ is a direct measure of goodness-of-fit $L(\pi,\pi_0)$ (Equation 2)—the degree to which π_0 is able to match the behavior of π. To see this, note that minimizing this KL is equivalent to performing maximum likelihood estimation for π_0 with π defining the 'true' underlying data distribution. Also observe that RNN_{π} is trained on-policy and RNN_{π_0} is trained off-policy from a buffer of experience collected by the control policy. We can view this as the control policy actively learning via trial and error in the world, while intuitively, within a multitask context, the default policy is trained to capture the behavior of the control policy on each task. The default policy thereby learns an 'average' of the behaviors required to perform well on each task. However, when only a few tasks have been observed, the default policy can 'overfit' to the behaviors learned on those initial tasks. This can be problematic, as if future tasks differ, the default policy could misguide the learning process for the control policy (see above). The VDO complexity regularization forces the default policy network to simplify, preventing overfitting and facilitating generalization.

Architecture details The LSTM dynamics were governed by the following standard equations:

$$\begin{aligned}
i_t &= \sigma(W_{xi}x_t + W_{hi}h_{t-1} + b_i)\\
f_t &= \sigma(W_{xf}x_t + W_{hf}h_{t-1} + b_i)\\
o_t &= \sigma(W_{xo}x_t + W_{ho}h_{t-1} + b_o)\\
c_t &= f_t \odot c_{t-1} + i_t \odot \tanh\left(W_{xc}x_t + W_{hc}h_{t-1} + b_c\right)\\
h_t &= o_t \odot \tanh(c_t),
\end{aligned}$$

where i_t, f_t, o_t, c_t, h_t are the input gate, forget gate, output gate, cell state, and hidden state at time t, respectively, $\sigma(x) = 1/(1+\exp(-x))$ is the sigmoid function, and $\odot$ denotes element-wise multiplication. In order to assess the degree to which the default policy learned to ignore certain input features, an element-wise gating layer $\ell(x)$ was applied to the input:

$$\ell(x_t) = \sigma(\tau\omega) \odot x_t,$$

where τ was a hyperparameter fixed across simulations and ω was a learned vector of parameters with dimension equal to that of the input. As $\omega_d \to \infty$, the dth input feature is passed on to the layers above, while if $\omega_d \to -\infty$, the dth input feature is gated out. Importantly, gradients from the VDO loss (see above) did not flow into ω, only those from the distillation loss, so ω learned to gate features in or out that were already either being used or ignored by the network, rather than simply being ablated directly as the VDO penalty increased. We found that adding this gating layer did not affect the performance of the agent.

Training details In all simulations, the agent was updated using a learning rate of $\eta = 0.0007$, a value function loss weight of $\alpha_v = 0.05$, a policy KL weight of $\alpha = 0.1$, a discount factor of $\gamma = 0.9$, a gating layer coefficient of $\tau = 150$, and a VDO KL weight of $\beta = 1.0$ unless otherwise noted. All gradient updates were performed using the Adam optimizer [105]. To generate the plots in Fig 4, agents were trained on their respective simulations with VDO KL weight $\beta \in \{0.0, 0.1, 1.0, 10.0, 100.0\}$ for all tasks except the two-step task, for which $\beta \in \{1.0, 10.0, 100.0, 1000.0, 10,000.0\}$ was used. After training, the average KL between policies and average VDO complexity KL were computed over 100 trials with frozen weights. Total KL was computed as the sum of these two quantities, with the VDO KL scaled by $\beta = 100$ for the two-step task. Further simulation-specific details can be found below, and a summary of hyperparameter values for each task is provided in Table 1. All results were obtained by averaging over 8 random seeds, with shading on line plots denoting one unit of standard error.

Table 1. Hyperparameters for each task.

**Task**	η	α_v	α	β	γ	M	τ	# hid.	$N_{\pi_0 \text{buf.}}$
Navigation	7e-4	0.05	0.2	10.0	0.99	1	150	48	1e5
Stroop	1e-3	0.05	0.2	0.005	0.99	1	150	16	1e5
Dem. Stroop	7e-4	0.05	0.2	1.0	0.99	1	150	48	1e5
Two-step	7e-4	0.05	0.1	3/100*	0.9	N/A	150	48	N/A
O & D	1e-3	0.05	0.2	1.0	N/A	1	150	16	1e3
Cont. contr.	3e-4	0.05	0.1	1.0	0.99	128	150	256	1e6
Heuristics	3e-4	N/A	0.1	1.0	N/A	32	150	128	1e6

*The two-step task settings are described in greater detail in the text.

Generalization

Navigation In this experiment, the agent was trained on two tasks within the classic FourRooms environment [109], an 11×11 gridworld in which the available actions were $\{\texttt{up}, \texttt{right}, \texttt{down}, \texttt{left}\}$. The agent LSTMs in both tasks each had 48 hidden units. Input feature importance to RNN_{π_0} was tracked using an element-wise gating layer (see "Architecture Details"). The state input at each time step s_t was an 11-dimensional vector comprising the index of the agent's current state, a flattened 3×3 square representing the agent's immediate surroundings (specifically, a value of 0 indicated a free space and a value of -1 indicated a barrier), and the index of the current goal location. Agents were trained in the following way: at the start of each episode, a goal location was randomly sampled from a set of possible goal locations. Episodes terminated when the agent either reached the goal location or once 250 steps had elapsed. The agent updated its weights after each episode. In the first task (Fig 1B, 2C), training was broken into two phases. In the first phase, the goal for each episode was randomly sampled from a set of possible goal states which were arranged in a checkerboard pattern (see Fig 1B inset). The agent was trained in this setting for 40,000 episodes. In the second phase, the checkerboard pattern was inverted, so that candidate goal states from the first phase could no longer contain a goal and vice versa. The agent was trained in this phase until it was able to reach a second phase goal in the fewest possible number of steps. The MDL-C agent was compared against a standard regularized policy optimization (Standard RL) agent with the same design, except it did not have the complexity KL penalty on its RNN_{π_0}.

In the second task (Fig 2D), at the start of each episode, a goal location was randomly sampled as either the top left state with probability 90% or the bottom right

state with probability 10%, with the agent starting anywhere in the environment with uniform probability. The agent was trained for 30,000 episodes.

Continuous control In this setting, the agent was trained sequentially on tasks from the Walker domain from the DeepMind control suite [110]. In this multitask setting, tasks were sampled one at a time uniformly without replacement from the available tasks within the Walker domain, with π_0 conserved across tasks. For Walker (visualized in Fig 1C inset), these tasks are `stand`, `walk`, and `run`. Observations are 25-dimensional feature vectors, with 1 dimension encoding the height of the agent's center of mass, 14 dimensions encoding position, 1 dimension encoding reward, and 9 dimensions encoding the velocity of various components of the agent. The action space is a 6-dimensional continuous vector which directs the agent's joints. In `stand`, reward is granted in proportion to the agent's height, and in `walk` and `run` further reward is given for forward velocity. See [110] for further environment details. Performance results for the `run`, the hardest task in the Walker domain are plotted in Fig 1C, where k indicates the task round at which the task was sampled. Unlike in other simulations, here π was trained off-policy using *soft actor-critic* (SAC; [104]) in order to improve sample-efficiency. As baselines, we included standard SAC (see [104] for details) and a MDL-C agent without the VDO complexity KL cost (termed RPO). Agents were trained on each task for 250,000 environment steps. As k increases—in other words, as the agent has been trained on more tasks—MDL-C's performance improves substantially.

1 Simulation 1: executive control

Stroop task In the Stroop task, the agent must perform either "word-reading" (WR) or "color-naming" (CN) tasks across two different colors and two different words, totalling eight different possible stimuli: (red[WR], blue[WR], blue[WR], red[WR], red[CN], blue[CN], blue[CN], red[CN]), each presented to the agent as a three-dimensional vector $x_t =$ [`color`, `word`, `task`] with the following encodings: blue$\rightarrow -1$, red$\rightarrow +1$, CN$\rightarrow -1$, WR$\rightarrow +1$. The presentation frequencies were 20% for all WR stimuli and 5% for all CN stimuli. There were two possible actions, corresponding to -1 and $+1$. The agent received $+1$ reward when its action matched the appropriate value of the stimulus feature (e.g., if the task feature is -1 and the color feature is $+1$, the desired action is $+1$) and zero otherwise. In order to simulate reaction times (RTs), the input stimulus for a given trial was re-presented up to a maximum of 5 times to the agent until the entropy of the control policy $H[\pi] = -\sum_a \pi(a|x_t;\theta)\log\pi(a|x_t;\theta)$ dropped below a threshold $b = 0.5$, similar to the approach to modeling RTs used by [87, 88]. The RT for each trial was the number of presentations of the stimulus until a response was produced. After a response was generated, the trial ended. The agent was trained for 15,000 trials and each LSTM had 48 hidden units.

Demand avoidance stroop task In this task, the agent was presented with word-reading (WR) and color-naming (CN) trials, encoded as in the Stroop task described above, with each WR stimulus having a 20% chance of presentation on any given trial. CN trials were presented 20% of the time, but in this task CN trials consisted of two time steps. On the first time step, the agent was presented with the stimulus $[0, 0, -1]$ to indicate a CN trial. Its action at this stage served to select between two categories, referred to as *high-demand* and *low-demand*. If the agent selected the high-demand category, then in the second time step of the trial, a conflict stimulus was presented with a 90% chance and a congruent stimulus with a 10% chance. If the agent

selected the low-demand category, these probabilities were reversed. The agent shared the same settings as in the main Stroop task, and was also trained for 15,000 trials.

Interference stroop task In this case, the agent was first pre-trained on color-naming-only trials for 30,000 trials. It was then trained for 45,000 trials on word-reading. During this training phase, the agent's reaction time was evaluated on both CN and WR trials (in evaluation trials, the agent's weights were not updated). The agent architecture and environment set-up were the same as in the "standard" Stroop task above. One important characteristic of the Stroop task is that there are interaction effects between task and congruity—that is, at the outset of training, color interferes with shape more than shape interferes with color. This means that shape-naming conflict trials result in disproportionately higher RTs compared to shape-naming congruent trials compared to the difference between color-naming conflict trials and color-naming congruent trials. This relationship is then reversed at the end of training, with a greater relative increase for color-naming trials. We verified that this property held for MDL-C by running a one-way ANOVA test on the differences in RT between each trial type (e.g., color-naming conflict RT - color-naming congruent RT vs. shape-naming conflict RT - shape-naming congruent RT), with the interaction effect at 0 trials yielding $F = 11.3$ and $p = 0.005$ and the interaction effect at 44,000 trials yielding $F = 13.7$ and $p = 0.002$.

Zero-shot stroop task The agent was trained 8,000 trials in which the unneeded feature was zeroed out from the stimulus (i.e., the agent gets 3d inputs [color, word, task id], where -1 = blue, +1 = red in the first two dims, -1 = color-naming, +1 = word-naming for task id, and 0 = NULL in any location). So, the agent would see [-1, 0, -1] for a "blue" color-naming task. The stimulus distribution was uniform. During training, both MDL-C and 'Regular RL" (just a control policy RNN_π with otherwise identical hyperparameters) get to 100% accuracy. The agent is then evaluated on 100 trials with fixed weights in which the unneeded feature is included in the stimuli. The evaluation performance is the percent correct over 100 evaluation trials with fixed weights. To test the hypothesis that the improved performance of MDL-C is rooted in robustness to changes in inputs, we also measured the average KL between the policy distributions for each approach on masked inputs (like the ones on which they were trained) and on inputs with the missing feature included. Regular RL had a greater difference, indicating that responses were more effected by the out-of-distribution inputs.

Simulation 2: reward-based learning

Two-step task We use a variant of the two-step task based on the one used by [86] in which transition contingencies—in addition to reward contingencies—may switch. The task was changed in this way following the finding by [96] that when transition contingencies are fixed, a habit-like strategy in which second stage states which have recently yielded reward are directly mapped to actions in the choice stage can develop which closely matches the pattern of behavior expected of agents using planning. Additionally, the agent is provided with an input feature which indicates which transition contingency setting is currently active (an ingredient added to the task from [96] in order to restore the property that model-based and -free strategies yield the classical patterns shown in Fig 5C). To use this feature to inform its actions, the agent must compute a higher-complexity policy than if this feature is ignored, analogous to the difference between classifying inputs according to `XOR` versus `OR` logic. To be more precise, with two second stage states A and B and two actions a_L and a_R, we can have

either

$$\text{Setting } 0 = \begin{cases} p(A|a_L) = 0.8, & p(B|a_L) = 0.2 \\ p(A|a_R) = 0.2, & p(B|a_R) = 0.8 \end{cases}$$

$$\text{Setting } 1 = \begin{cases} p(A|a_L) = 0.2, & p(B|a_L) = 0.8 \\ p(A|a_R) = 0.8, & p(B|a_R) = 0.2 \end{cases}$$

In other words, in one setting a_L is likely to lead to A and a_R is likely to lead to B, and in the other, the reverse is true. The agent is shown a binary feature which indicates which transition setting the environment is in (however, it has to learn what this feature means through experience). More precisely, the state observation at each time step s_t is a 5-dimensional vector, with the first four dimensions comprising a one-hot encoding of the current position of the agent within the task (either `fixation stage`, `choice stage`, `A`, or `B`), with the final dimension a binary encoding of the current transition setting. The agent has three possible actions: a_L, a_R, and a_{fixate}, which the agent is required to produce in order to progress from the fixation stage to the choice stage. There are also two possible settings for the reward contingencies, with either A or B having a 90% chance of leading to reward, with the other state in either contingency having a 10% chance. The agent is trained for 16,000 episodes, where each episode consists of 100 trials. At the end of each episode, the agent networks' hidden states are reset and an update is performed via backprop. On any given trial, there is a 2.5% chance that the reward contingency switches and a 5% chance that the transition contingency changes. During training, we found it helpful to start with a 0% chance of reward contingency switches and linearly increase the probability to 2.5% over the first 2,000 episodes, as this helped the agent reliably learn the meaning of the transition setting feature. All other task settings and analysis details for stay probabilities and logistic regression are the same as in [86]. Importantly, in this task the default policy was trained online (but still off-policy) via full trajectories collected by the control policy, rather than via a buffer of (s, a, r, s') tuples. This is because the full episode history is required to effectively meta-learn, as demonstrated by [86,111]. The hyperparameter settings used to generate the plots in Fig 5(D-F) were identified after an initial grid search with eight random seeds per $(\alpha, \beta, RewardScale)$ tuple with $\alpha \in \{0.05, 0.1, 0.2\}$, $\beta \in \{0.1, 1.0, 3.0, 5.0, 10.0, 100.0\}$, $RewardScale \in \{0.5, 0.75, 1.0\}$ and further confirmed by an additional eight random seeds, for a total of 16. The 'classic' MB-MF patterns were obtained with $(0.1, 100.0, 1.0)$ and the mixed patterns were observed with $(0.2, 3.0, 0.75)$. To further support the mixed MB-MF-ness of the response pattern in Fig 5F, we performed Wilcoxon signed-rank tests between the average of the rewarded, common and unrewarded, uncommon responses and the average of the rewarded, uncommon and unrewarded, common responses as a measure of model based-ness, and between rewarded, common and rewarded, uncommon responses and unrewarded, common and unrewarded, uncommon responses as a measure of model free-ness. The response patterns for both the control and default policies were statistically significant ($p = 0.012$) for both model-based and model-free behavior. The agent's LSTMs had 48 hidden units each.

Perseveration In this experiment, the agent was trained on the drifting two-armed bandit task from [119]. In this task, trials consist of a single time-step in which the agent has two possible actions, with the probability of reward for each arm evolving with a Gaussian random walk. Specifically, if the probability of being rewarded by choosing a given action on trial t is P_t, then the probability of being rewarded for choosing that arm on the next trial is sampled from the distribution $P_{t+1} \sim \mathcal{N}(P_t, 0.15^2)$. The agent

either receives a reward of 1 or 0, and is trained for 3,000 trials. In this case, each RNN had 5 hidden units. After training, logistic regression is performed to predict the agent's behavior on a given trial, with the regressors being the choice made at each time-step (± 1), the whether a reward was given at each time-step (± 1), and their product. A high regression weight for previous choices indicates a tendency to perseverate, a high weight for the outcome/reward indicates that the agent is influenced by whether it was rewarded at each step independent of its previous choices, and a high regression weight for their product indicates that the agent is influenced by choices that led to rewards (reward-seeking behavior).

Omission and contingency degradation We use the same task set-up as [58]. As in [58], in order to model the effect of overtraining on the agent's sensitivity to omission of reward, the agent was first trained on a two-armed bandit task in which action 1 ("lever press") led to a reward of 1 with 50% probability and action 2 ("leisure") resulted in a reward of 0.1 100% of the time. It was then trained for 750 trials on a modification of the task in which reward was never delivered for lever pressing and in which leisure resulted in a reward of 0.1 half the time and a reward of 1.1 half the time. The agent's lever-pressing probability $P(\text{lever press})$ was then measured at the end of the second training phase. This probability was plotted against the number of trials T for which the agent was trained on the first phase, where $T \in [100, 200, 300, \ldots, 2000]$. The contingency degradation variant of this task was exactly the same, except that the leisure action always resulted in a reward of 0.1 in the second phase.

2 Simulation 3: judgment and decision-making

Heuristics We use the same experimental setting as [62]. Briefly, the agent is meta-trained on a series of randomly generated paired comparison tasks with continuous input features x in which it must predict which of two presented feature vectors $x_t = (x_t^A, x_t^B)$ is associated with a higher value of an unobserved scalar criterion $y_t = (y_t^A, y_t^B)$. More precisely, for each task i, there is an underlying linear relationship between features and the unobserved criterion:

$$y_{t,A} = w_i^\top x_{t,A} + \epsilon_{t,A};$$
$$y_{t,B} = w_i^\top x_{t,B} + \epsilon_{t,B},$$

where $\epsilon_{t,A}, \epsilon_{t,B} \sim \mathcal{N}(0, \sigma^2)$, with σ^2 a fixed variance and $w_i \in \mathbb{R}^4$. An ideal observer model then expresses the probability that $y_A > y_B$ as

$$p(y_A > y_B | x, w_i) = p(C = 1 | x, w_i) = \Phi\left(\frac{w_i^\top x}{\sqrt{2}\sigma}\right), \quad (3)$$

where $\Phi(\cdot)$ is the cumulative distribution function of a standard Gaussian distribution and $C \in \{0, 1\}$ is a binary random variable which evaluates to 1 when $y_A > y_B$ and 0 otherwise. Task feature weights w_i are randomly generated from a standard normal distribution, and the agent is meta-trained to estimate a posterior distribution over w with minibatches of 32 tasks and each task being presented to the agent for 10 trials. The reward for a given trial can be modeled as the log likelihood: $p(C_t | x_t, \phi_t)$ For a more detailed description of the training process, see [62]. The control network RNN_π produces the parameters (mean μ_t and variance Ψ_t) of an approximate Gaussian posterior over w_i, which is then integrated into a predictive distribution for classification:

$$p(C_{t+1} | x_{t+1}, \phi_t, \Theta) = \int p(C_{t+1} | x_{t+1}, w) q(w; \phi_t) \, dw$$

where and $\phi_t = \{\mu_t, \Psi_t\}$ are the parameters of the approximate Gaussian posterior q over parameters w. The conditional distribution is as above

$$p(C_{t+1} = 1 | x_{t+1}, w) = \Phi\left(\frac{w^\top x_t}{\sqrt{2}\sigma}\right).$$

The default network RNN_{π_0} also produces parameters ϕ_t^0 of a Gaussian q_0 and is trained to minimize $D_{KL}(q(w, \phi_t)||q_0(w, \phi_t^0))$ in addition to the VDO complexity KL weighted by β (see "Default Policy" details above).

To test the emergence of heuristics, we use the task variant from [62] in which there is a known ranking of input features, which classically induces a form of one-reason decision-making termed "take the best" (TTB), wherein subjects make decisions based on the top-ranked feature which differs between two inputs. To measure the emergence of such a heuristic in artificial agents, [62] use the *Gini coefficient* G [108] measured over the feature weights w, defined below:

$$G(w) = \frac{\sum_{i=1}^{d} \sum_{j=1}^{d} |w_i - w_j|}{2d \sum_{i=1}^{d} w_i}.$$

The Gini coefficient can be thought of as a measure of inequality among feature weightings, so that it tends to 1 when one feature grows in importance compared to the others, and tends to 0 as all feature weights w_i converge to the same value. As a means of probing the effect of reducing the relative cost of employing a compensatory strategy (Fig 6D), we reduced the weighting on the KL between the default and control policies, setting $\alpha = 0.01$. This effectively lowers the penalty for deviation in behavior from the capacity-limited policy.
